# Spica Prunellae attenuates ER stress via GRP78/IRE1α/JNK pathway in CCL_4_-induced acute liver injury in mice

**DOI:** 10.3389/fphar.2026.1909409

**Published:** 2026-10-05

**Authors:** Yanjie Wang, Jiaping Hao, Jinfeng Yang, Qiaomei Dai, Zhimin Chen, Feifei Han, Yiwen Zhang, Huijun Zhang

**Affiliations:** 1 School of Food and Biological Engineering, Hezhou University/Guangxi Key Laboratory of Healthcare Food Science and Technology, Hezhou, China; 2 School of Basic Medicine, Heilongjiang University of Chinese Medicine, Harbin, China; 3 School of Materials and Chemistry, Hezhou University, Hezhou, China

**Keywords:** CCl_4_, endoplasmic reticulum stress, GRP78, IRE1α, JNK, Spica Prunellae

## Abstract

**Objective:**

To evaluate the effects of Spica Prunellae (SP) on CCl_4_-induced acute liver injury (ALI) in mice and its regulation of the endoplasmic reticulum stress pathway.

**Methods:**

In the toxicity test, ICR mice were administered SP decoction (2 g/kg, crude herb/mouse weight) by gavage for 10 days. Liver and kidney tissues were collected for H&E staining, and serum levels of liver function (AST, ALT) and renal (BUN, SCR) function indicators were measured. After confirming safety, 48 ICR mice were randomly assigned to normal, model, high-, medium-, and low-dose SP, and positive control groups (*n* = 8 per group). All mice, except the normal group, received a 10% CCl_4_/peanut oil intraperitoneal injection to induce ALI. The normal and model groups received saline; the positive control group received bifendate; and the SP groups received corresponding SP doses by daily gavage for 10 days. Body weight, liver, spleen, and thymus indices were recorded; ELISA measured serum ALT and AST, and liver MDA and SOD; liver histopathology was assessed by H&E staining; hepatocyte ultrastructure was examined *via* transmission electron microscopy; and ER stress-related proteins (GRP78, IRE1α, and JNK) expressions were determined by Western blot.

**Results:**

SP caused minimal damage to hepatic and renal tissues in mice at 2 g/kg, with no significant changes in liver (ALT, AST) or renal (BUN, SCR) function indicators (*p* > 0.05). In the ALI model vs. model group, SP-treated groups demonstrated improved organ indices (*p* < 0.05), reduced serum ALT and AST levels (*p* < 0.05), increased SOD levels (*p* < 0.05), and significantly decreased MDA levels (*p* < 0.01). Histopathological analysis revealed clear attenuation of liver tissue damage, with mild nuclear deformation. Endoplasmic reticulum degranulation was alleviated, showing an organized arrangement and preserved cellular structure. Compared to the model group, GRP78 and IRE1α expression were significantly declined in the positive control and SP-treated groups (*p* < 0.01, *p* < 0.05), and JNK expression was downregulated (*p* < 0.05).

**Conclusion:**

These findings suggest the involvement of the GRP78/IRE1α/JNK signaling pathway in the hepatoprotective action of SP against CCl_4_-induced ALI.

## 1 Introduction

The liver, as the primary metabolic organ in the human body, plays a central role in the metabolism of proteins, lipids, and carbohydrates. It is highly susceptible to injury caused by alcohol, drugs, and viral infections (Yuan et al., 2018; Breu et al., 2019). Globally, liver diseases account for over two million deaths annually (Zhu S et al., 2024), with acute liver injury (ALI) representing a major health concern (Billerbeck et al., 2017). Chronic or persistent liver damage can progress to severe conditions such as fibrosis, cirrhosis (Taru et al., 2024), and hepatocellular carcinoma, ultimately leading to liver failure (Stravitz and Lee, 2019).

Endoplasmic reticulum (ER) stress has recently gained significant attention in the prevention and treatment of liver injury, as it contributes to hepatic damage through multiple pathways. Glucose-regulated protein 78 (GRP78), a marker of ER stress, has been shown to mediate inflammatory responses and mitigate abnormal liver function and histopathological damage (Song et al., 2023). GRP78 can activate JNK *via* its downstream effector IRE1α, and the JNK-mediated pathway is critically involved in processes such as cellular stress responses, ER stress, apoptosis, and autophagy (Shi et al., 2019; Gao et al., 2021).

Traditional Chinese medicine (TCM) has effective therapeutic strategies for liver diseases and a long history of clinical experience. In TCM, ALI treatment is provided based on the symptoms and signs of the patient. Several compound prescriptions and herbs have been shown to clinically alleviate liver injury and improve liver function indicators (Huang et al., 2024; Wang et al., 2025). Spica Prunellae (SP) is a TCM herb with anti-inflammatory and detoxifying effects. Being pungent, bitter, and cold in nature, SP is typically used as dried fruit spikes, and it acts on the liver and gallbladder meridians. SP functions include reducing liver inflammation, improving eyesight, and softening and resolving masses (Chinese Pharmacopoeia Commission, 2020). SP contains various bioactive compounds, including polysaccharides, flavonoids, phenolic acids, sterols, and coumarins, which show antimicrobial, immunomodulatory, anticancer, cardioprotective, antiallergic, anti-inflammatory, and antihypertensive effects (Mir et al., 2022). Several key components in SP have therapeutic effects on ALI by improving liver function, reducing hepatocellular degeneration and necrosis, and enhancing anti-inflammatory and antioxidant capacities (Zhang et al., 2012; Wan et al., 2016).

CCl_4_-induced liver injury is closely associated with endoplasmic reticulum stress (ERS) (Chen et al., 2015; Li et al., 2018), and inhibition of ERS can attenuate CCl_4_-induced liver damage. The present study aims to investigate the effects of SP on acute liver injury (ALI) induced by CCl_4_ in mice and to elucidate its mechanism of action in regulating endoplasmic reticulum stress (ERS), particularly through modulation of the GRP78/IRE1α/JNK signaling pathway.

## 2 Materials and methods

### 2.1 Study animals

Forty-eight healthy SPF-grade ICR mice (weighing 18–22 g with equal numbers of males and females) were obtained from Liaoning Changsheng Biotechnology Co., Ltd. The mice were housed in an environment with a temperature of 20 °C–25 °C, 40%–60% relative humidity, and a 12-h light/dark cycle. They had unrestricted access to food and water, and all experimental procedures were conducted after a 3-day acclimation period. The study protocol was approved by the Ethics Committee for Medicine and Experimental Animals at Heilongjiang University of Chinese Medicine.

### 2.2 Chemicals and reagents

CCl_4_ (C805332)was procured from Shanghai Macklin Biochemical Technology Co., Ltd., bifendate pellets were sourced from Beijing Union Pharmaceutical Factory, and SP was provided by the First Affiliated Hospital of Heilongjiang University of Chinese Medicine. Aspartate aminotransferase (AST) (c010-2–1), alanine aminotransferase (ALT; c009-2–1), blood urea nitrogen (BUN; C013-2–1), serum creatinine (SCR; C011-2–1), malondialdehyde (MDA; A003-1–2), and superoxide dismutase (SOD) (A001-1–2) assay kits were obtained from Nanjing Jiancheng Bioengineering Institute. BCA protein quantification/concentration kits, SDS-PAGE gel rapid preparation kits, ultra-sensitive ECL luminescent solution, and pre-stained rainbow protein markers were obtained from Dalian Meilun Biotechnology Co., Ltd. H&E staining kits and alcohol-soluble H&E staining solution were supplied by Beijing Biotopped Technology Co., Ltd., while 1% acidic ethanol differentiation solution was purchased from Fuzhou Phygene Biotechnology Co., Ltd. SDS-PAGE electrophoresis buffer (Tris-Gly, 10×), Western blot transfer buffer, washing buffer, and transfer filter paper were acquired from Beyotime Biotechnology Institute. Rabbit Anti-JNK antibody (bs-10562 R), Rabbit Anti-GPR78 Antibody (bs-1219 R), Rabbit Anti-IRE1α antibody (bs-8680 R), β-Actin Polyclonal Antibody (bs-0061 R), and goat anti-rabbit IgG H&L/HRP antibody (bs-40295G-HRP) were provided by Beijing Bioss Biotechnology Co., Ltd.

### 2.3 Instrument

TGL16 benchtop high-speed refrigerated centrifuge (Changsha Yingtai Instrument Co., Ltd.), FA124 electronic balance (Shanghai Sunny Hengping Scientific Instrument Co., Ltd.), DGX-9143B-4 electric thermostatic drying oven (Shanghai Fuma Laboratory Equipment Co., Ltd.), E.G.,1150H fully automatic embedding machine (Leica, Germany), HM355 S histopathological microtome (Thermo Fisher, Uinted States), KD-TH1 biological tissue baking and drying machine (Jinhua Kedee Instrument Equipment Co., Ltd.), Infinite M200PRO microplate reader (Tecan, Switzerland), electrothermal constant temperature water bath (Shanghai Yiheng Scientific Instrument Co., Ltd.), H-7700 transmission electron microscope (TEM; Hitachi, Japan), electrophoresis apparatus, electrophoresis tank, and electrotransfer apparatus (Bio-Rad, Uinted States), and STS-3 decolorization shaker (Shanghai Qite Analytical Instrument Co., Ltd.) were used in this study.

### 2.4 Preparation of SP decoction

SP was soaked in water for 2 h, followed by decoction with 30 volumes of water for 1.5 h. Following, 15 volumes of water were added, and the mixture was decocted over low heat for 1 h. The decoction was filtered through four layers of gauze, and the two filtrates were combined and allowed to stand overnight. The supernatant was collected the following day, while the precipitate was centrifuged at a speed of 3,000 rpm for a duration of 20 min to obtain an additional supernatant. The supernatants were combined and further decocted and concentrated to achieve a crude drug concentration of 2 g/mL.

Bifendate pellets were ground using a mortar, fully dissolved in double-distilled water, and prepared as a 15 mg/mL suspension, which was mixed thoroughly before gavage. All prepared solutions were aliquoted into 5 mL centrifuge tubes and stored at −20 °C until further use.

### 2.5 Acute toxicology experiment

Following 3 days of acclimatization, 16 specific pathogen-free (SPF) ICR mice (equal numbers of males and females) were randomly assigned into two groups (n = 8 per group): a normal control group and an SP-treated group. The SP group received SP extract (2 g crude drug/kg body weight) *via* oral gavage, while the control group received an equivalent volume of normal saline. Following 10 days of continuous administration, recording of the body weight was carried out, and samples of blood were collected *via* retro-orbital sinus sampling. The mice were then humanely euthanized by cervical dislocation. Liver and kidney tissues were harvested for pathological examination using H&E staining, and serum samples were collected for the assessment of liver and kidney function markers.

#### 2.5.1 Detection of serum hepatic and renal function indicators

Blood samples were collected *via* retro-orbital sinus puncture and centrifuged at 3,000 rpm for 15 min at 4 °C to obtain serum. All biochemical analyses were performed using commercial assay kits (Nanjing Jiancheng Bioengineering Institute, China), according to the manufacturer’s protocols. For ALT and AST analysis, 20 μL substrate solution was added to each well, followed by 5 μL serum or control solution and incubation at 37 °C for 30 min. Then, 20 μL of 2,4-dinitrophenylhydrazine was added and incubated for 20 min at 37 °C. Finally, 200 μL of 0.4 mol/L NaOH was added, and absorbance was measured at 510 nm after 15 min at room temperature.

For BUN determination, 20 μL distilled water, standard (10 mmol/L), or serum samples were mixed with 250 μL buffer enzyme solution and incubated at 37 °C for 10 min. This was followed by the addition of 1,000 μL phenol chromogen and 1,000 μL alkaline hypochlorite, with a further 10 min incubation at 37 °C. Absorbance was read at 640 nm.

For SCR measurement, 6 μL serum, standard, or distilled water was mixed with 180 μL enzyme solution A and incubated at 37 °C for 5 min. Absorbance (A_1_) was recorded at 546 nm. Then 60 μL enzyme solution B was added, incubated for another 5 min, and absorbance (A_2_) was measured. SCR concentration was calculated based on the difference in absorbance (A_2_ − A_1_).

#### 2.5.2 H&E staining for pathological assessment of liver and kidney tissues

Paraffin sections of liver and kidney tissues were prepared using standard procedures. Sections were immersed in xylene for 7 min, followed by rehydration through a graded ethanol series (100%, 95%, 75%, and 70%) for 3 min each, with each step repeated twice. Sections were rinsed under running water for 10 s, stained with hematoxylin for 7 min, and rinsed again for 1 min. Differentiation was performed using 1% hydrochloric acid-ethanol solution for 5 s, followed by rinsing under running water for 3 s. Sections were then treated with 0.2% ammonia solution for 10 s and rinsed for 1 min. The following sections were immersed in 95% ethanol for 1 min, treated with eosin for 5 min, and rinsed under running water for 1 min. Dehydration was carried out through 95% ethanol and anhydrous ethanol (3 min each, repeated twice), followed by clearing in xylene for 5 min. The sections were sealed with neutral gum, air-dried, and stored for further analysis.

### 2.6 Model establishment, grouping, and sample collection

Following a 3-day adaptive feeding period, ICR mice were stratified by sex and body weight, and then randomly assigned to six groups: normal control, model, high-dose SP, medium-dose SP, low-dose SP, and positive control (bifendate). A total of 60 mice were initially enrolled (10 per group). After the experiment, data from accidental deaths and outliers identified by the Q-test (Grubbs' test, defined as values deviating more than two standard deviations from the group mean) were excluded from the final analysis. The final analysis was therefore performed on 8 mice per group n=8 .

The normal control and model groups received normal saline (10 mL/kg) by gavage, while the positive control group received bifendate suspension (150 mg/kg). The high-, medium-, and low-dose SP groups were administered 2, 1, and 0.5 g/kg of SP, respectively, once daily for 10 consecutive days. Body weight was recorded 2 h after the final administration. All groups except the normal control group received a single intraperitoneal injection of 10% CCl_4_ in peanut oil (10 mL/kg) to induce acute liver injury. Mice were then fasted with free access to water. After 24 h, body weight was recorded, blood samples were collected via retro-orbital sinus puncture, and mice were euthanized by cervical dislocation. Liver, spleen, and thymus tissues were rapidly excised, weighed, and preserved for further analysis.

After 10 days of administration, body weight was recorded 2 h after the final dose. All groups, except the normal control group, were injected (*i.p*) with a 10% CCl_4_/peanut oil mixture (10 mL/kg) to induce ALI. Mice were then fasted with free access to water. After 24 h, the body weight was recorded, samples of the blood were collected, and mice were euthanized as described above. Liver, spleen, and thymus tissues were rapidly excised and preserved for further analysis.

### 2.7 Organ index

Excised liver, spleen, and thymus tissues were rinsed repeatedly with physiological saline at 4 °C, blotted dry with filter paper, and weighed. The organ index was calculated using the following formula (Equation 1):

$$\text{Organ index }\left(\%\right)=\frac{Absolute\;organ\;weight}{Body\;weight\;before\;euthanasia}\times 100\%$$ (1)

### 2.8 Biochemical analysis

#### 2.8.1 Serum AST, ALT, BUN, and SCR

Serum was collected as described in the above section. Measurements of AST and ALT were performed by ELISA according to the kit instructions. OD values were recorded at 510 nm, and the concentrations of AST and ALT were calculated. BUN was determined by measuring OD at 640 nm, whereas SCR was assessed at 546 nm. The experimental procedure followed the protocol described in Section 2.5.1.

#### 2.8.2 Liver MDA and SOD level

Fresh mouse liver tissue (0.1 g) was weighed and homogenized on ice with 9 volumes of homogenization buffer (weight [g]: volume [mL] = 1:9) for the preparation of a tissue homogenate (10%) using a glass tissue grinder. The homogenate was centrifuged at 3,500 rpm for 10 min at a temperature of 4 °C, and the supernatant was collected. The concentration of protein was determined using the BCA method. MDA and SOD levels were measured through an ELISA kit by following the manufacturer’s instructions, and OD was recorded at 550 nm.

### 2.9 Histopathology

Fixation of the fresh liver tissue was carried out with 4% paraformaldehyde for 24 h, washed, dehydrated through graded ethanol solutions, cleared with xylene, and embedded in paraffin. Tissue sections were cut to a thickness of 3 μm. Sections with intact tissue and smooth surfaces free of knife marks were selected and mounted on a tissue slicer. Sections were spread at 42 °C and then placed in a 60 °C incubator for 1 h. After hematoxylin staining and development of a blue color, slides were differentiated using a differentiation solution, followed by re-staining with hematoxylin blue. Cytoplasm was stained with an eosin solution. Sections were dehydrated through graded ethanol, cleared with xylene, sealed with neutral resin, air-dried, and examined under a light microscope, with representative images captured. For histopathological quantification, ImageJ software (version 1.54f, National Institutes of Health, Uinted States) was used to analyze the representative images from each group. The necrotic area was measured by tracing the boundary of necrotic regions using the Freehand selection tool, and the percentage of necrotic area was calculated as (necrotic area/total field area) × 100%. Each image was measured three times to obtain an average value, and the data are presented as mean ± SD. All images were captured under identical acquisition settings, and all measurements were performed by two independent observers blinded to the experimental groups.

### 2.10 Transmission electron microscopy (TEM)

Fresh mouse liver tissue (∼1 mm^3^) was collected and processed on ice. Samples were fixed, dehydrated through graded ethanol, cleared, embedded, and polymerized, followed by overnight incubation in a 60 °C oven. Ultrathin sections (60 nm) were prepared using an ultramicrotome and subjected to double staining with uranyl acetate and lead citrate. The prepared sections were examined under a TEM, and representative images were captured.

### 2.11 Western blot analysis

Liver tissue (0.1 g) from each group was homogenized on ice in lysis buffer using a glass grinder. The samples were then centrifuged to obtain the supernatant, and protein levels were measured using the BCA assay. The loading amount was 20 µg total protein per lane. The samples were denatured by boiling for 5 min. Proteins were separated by SDS-PAGE, with 5 μL of protein marker loaded alongside samples. Electrophoresis was performed at 80 V until proteins entered the separating gel, then at 140 V until reaching the gel bottom. The separated proteins were blotted to a membrane at 100 V for 2 h in an ice bath. Membranes were blocked in 5% skimmed milk overnight at 4 °C, then treated with primary antibodies (GRP78, IRE1α, JNK) at a 1:1,000 dilution for 2 h on a shaker. β-actin was used as the internal control at 1:5,000 dilution. After three washes with 1×TBST (5 min each), the incubation of the membranes was carried out with secondary antibody for 90 min on a shaker, washed three times (5 min each). Signals were developed using an enhanced chemiluminescence (ECL) reagent for 1–2 min at room temperature. Blots were visualized using a UVITEC Cambridge gel imaging system, and band intensities were analyzed using ImageJ software (version 1.54f, National Institutes of Health, Uinted States). Band intensities were normalized to β-actin and expressed as relative fold change compared with the control group. All Western blot experiments were performed in triplicate (*n* = 3 independent biological replicates per group).

### 2.12 Statistical analysis

All data are expressed as the mean ± standard deviation. Before statistical analysis, the data were evaluated for normal distribution and homogeneity of variance to ensure the suitability of parametric testing. Group differences were then assessed using one-way ANOVA, and pairwise comparisons were conducted using the LSD post-hoc test because the comparisons were pre-planned (model group vs. Each treatment group). LSD is appropriate when the number of comparions is limited and clearly defined *a priori*, and it offers higher statistical power for detecting differences in planned comparisons compared with conservative tests such as Tukey or Bonferroni. Because the comparisons were pre-planned (model group vs. Each treatment group). LSD is appropriate when the number of comparions is limited and clearly defined *a priori*, and it offers higher statistical power for detecting differences in planned comparisons compared with conservative tests such as Tukey or Bonferroni. Differences were considered statistically significant when the *p*-value was less than 0.05.

## 3 Results

### 3.1 Effects of SP on serum biochemistry in toxicological evaluation

Mice administered SP (2 g/kg) by oral gavage for 10 days maintained normal physiological status, including a glossy coat, normal activity, and stable appetite and bowel function, with no observable abnormalities. No significant differences were detected in serum hepatic (AST and ALT) (Figure 1a) or renal (BUN and SCR) markers (Figure 1b) between the normal group and the SP-treated group (*p* > 0.05). These findings indicate that repeated administration of high-dose SP did not impair hepatic or renal function in normal mice.

**FIGURE 1:**
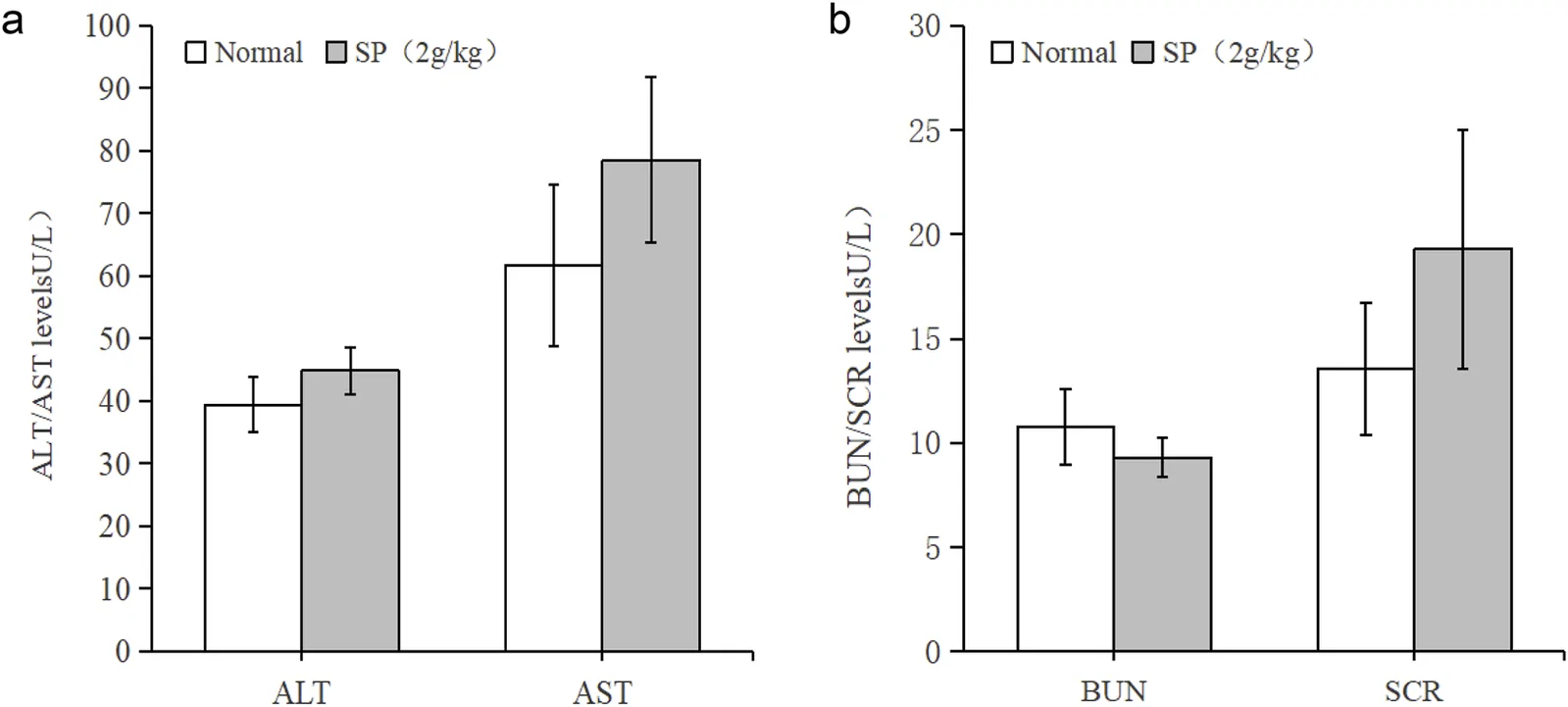
Effects of SP on hepatic and renal function parameters during toxicological evaluation. **(a)** Serum AST and ALT levels. **(b)** Serum BUN and SCR levels. No statistically significant differences were observed between the experimental and control groups, suggesting that SP did not induce detectable hepatic or renal dysfunction and was well tolerated at the administered dose.

### 3.2 Histopathological assessment in toxicological evaluation

Histopathological evaluation using H&E staining showed normal liver architecture in the normal group, characterized by well-organized hepatocytes, intact hepatic lobules, and normal sinusoidal structures. (Figure 2a). No evidence of cellular swelling, degeneration, or necrosis was observed. The SP-treated group (2 g/kg) showed comparable hepatic morphology, with preserved cellular structure and no pathological alterations (Figure 2c). Renal histology in the normal group demonstrated intact glomeruli and renal tubules with normal cellular morphology and no signs of inflammation or degeneration (Figure 2b). Similar observations were observed in the SP-treated group, with no detectable structural abnormalities (Figure 2d). These results suggest that SP administration alone does not induce histopathological changes in liver or kidney tissues.

**FIGURE 2:**
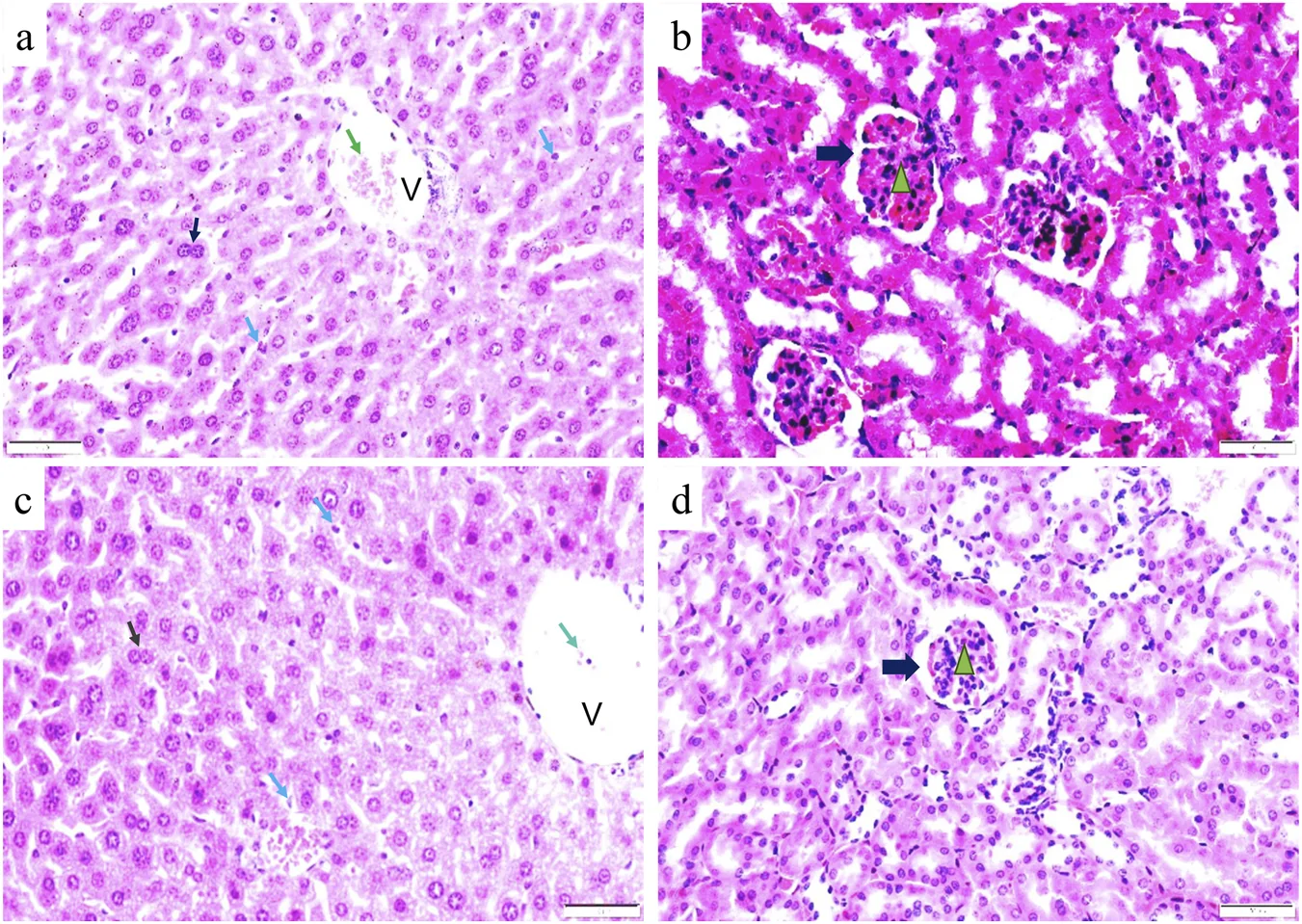
Effects of SP on hepatic and renal tissues during toxicological evaluation. Representative H&E-stained sections of liver **(a,c)** and kidney **(b,d)** from the normal **(a,b)** and SP-treated **(c,d)** groups at ×400 magnification. In the liver sections **(a,c)**: green arrows indicate red blood cells (RBCs); “V” denotes the central vein; blue arrows indicate binucleated hepatocytes; and black arrows indicate Kupffer cells. In the kidney sections **(b,d)**: black arrows point to Bowman’s capsule; and green triangles indicate the glomeruli. No pathological lesions were observed in any of the sections.

### 3.3 Effects of SP on organ indices in CCl_4_-induced ALI

As compared to the model group, the positive control group, and all SP-treated groups showed a significant reduction in liver index (Figure 3a) and spleen index (Figure 3b) (*p* < 0.01). A significant difference in the thymus index was observed between the normal group and the model group (Figure 3c) (*p* < 0.05). Relative to the normal group, the model group displayed marked increases in liver and spleen indices (*p* < 0.01), indicating hepatomegaly and splenic enlargement. The elevation in liver index suggests pathological alterations such as congestion and edema following CCl_4_ exposure. These observations confirm the successful establishment of the ALI model and indicate that SP treatment mitigates organ index alterations associated with CCl_4_-induced injury.

**FIGURE 3:**
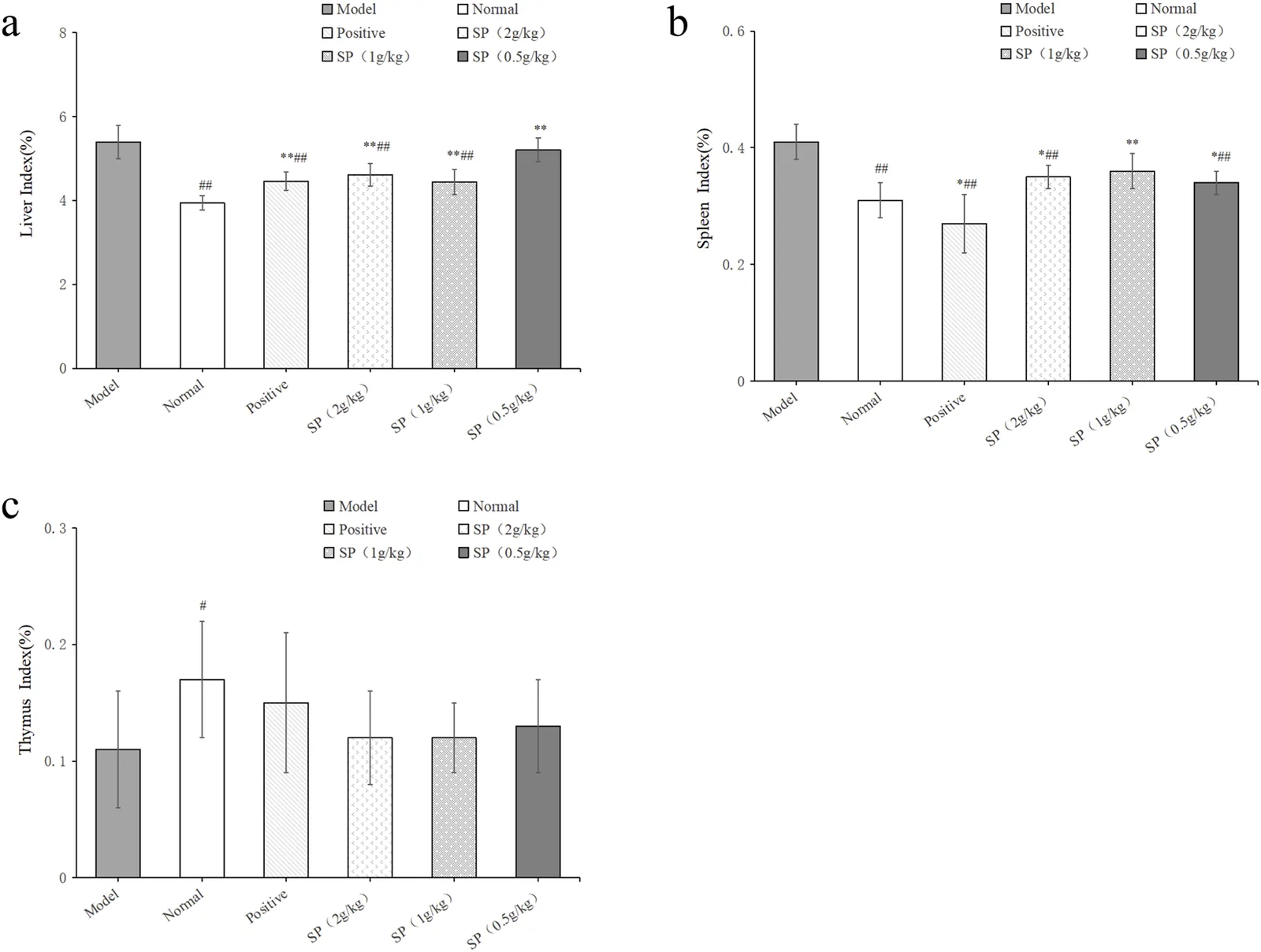
Effects of SP on organ indices in CCl_4_-induced ALI mice. **(a)** Liver index. **(b)** Spleen index. **(c)** Thymus index. ^##^ *p* < 0.01, ^#^ *p* < 0.05 vs. model group; **p < 0.01, *p < 0.05 vs. normal group.

### 3.4 Effects of SP on liver function markers in CCl_4_-induced ALI mice

Serum ALT and AST levels were significantly elevated in the model group vs. the normal group (*p* < 0.01), confirming successful induction of liver injury. Administration of bifendate pellets or SP resulted in significant reductions in serum ALT (Figure 4a) and AST (Figure 4b) vs. *the* model group (*p* < 0.01, *p* < 0.05). These results indicate that SP mitigates CCl_4_-induced hepatic injury as reflected by reduced serum liver enzyme levels.

**FIGURE 4:**
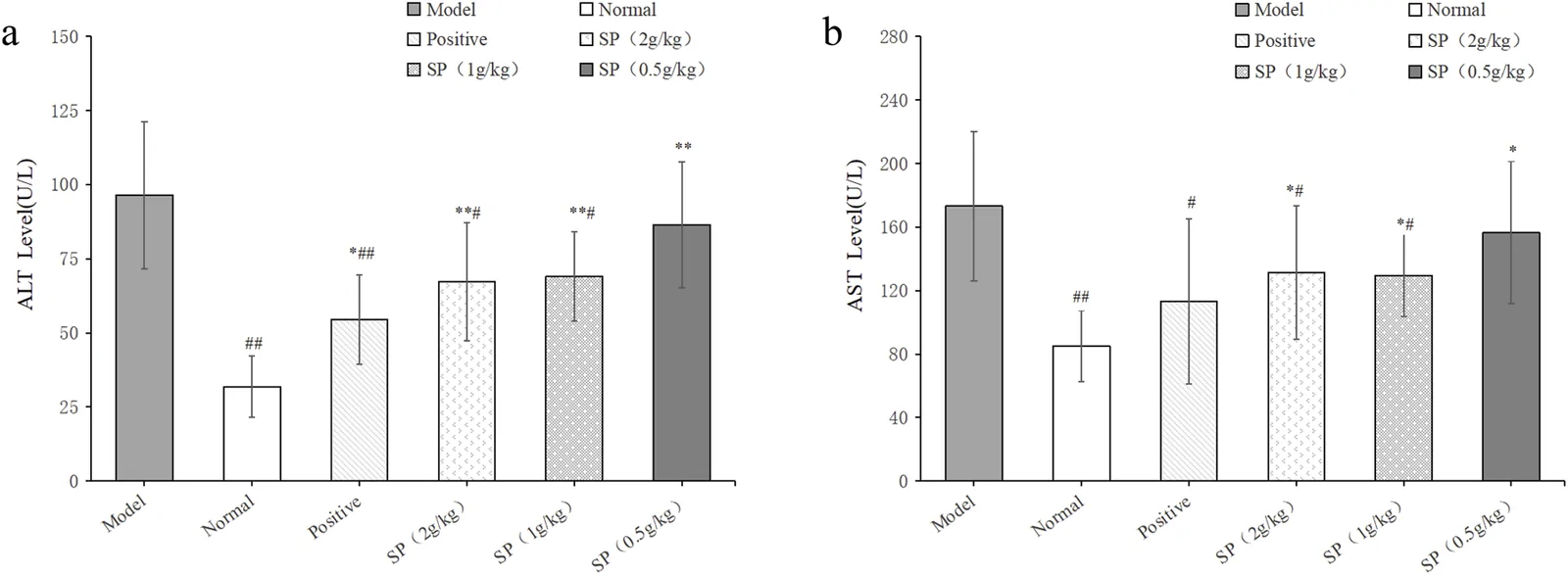
Effects of SP on liver function markers in CCl_4_-induced ALI mice. **(a)** Serum level of ALT. **(b)** Serum level of AST. ^##^ *p* < 0.01, ^#^ *p* < 0.05 vs. model group; **p < 0.01, *p < 0.05 vs. normal group.

### 3.5 Effects of SP on hepatic histopathology in CCl_4_-induced ALI mice

H&E staining showed that hepatocytes in the normal group were regularly arranged, with intact hepatic lobule and sinusoidal structures, and no evidence of swelling, degeneration, or necrosis (Figure 5b). In the model group, hepatocytes showed disorganization, swelling, and degeneration, accompanied by extensive inflammatory cell infiltration, necrosis, and marked sinusoidal congestion (Figure 5a). Treatment with bifendate or SP alleviated liver injury to varying degrees. In the positive control group, cellular degeneration, necrosis, and inflammatory infiltration were markedly reduced, and tissue architecture was relatively restored (Figure 5c). In SP-treated groups (Figures 5d–f), hepatocyte damage and swelling were attenuated, inflammatory infiltration and necrosis were reduced, and tissue structure approached normal, with the effect being more significant at higher doses.

**FIGURE 5:**
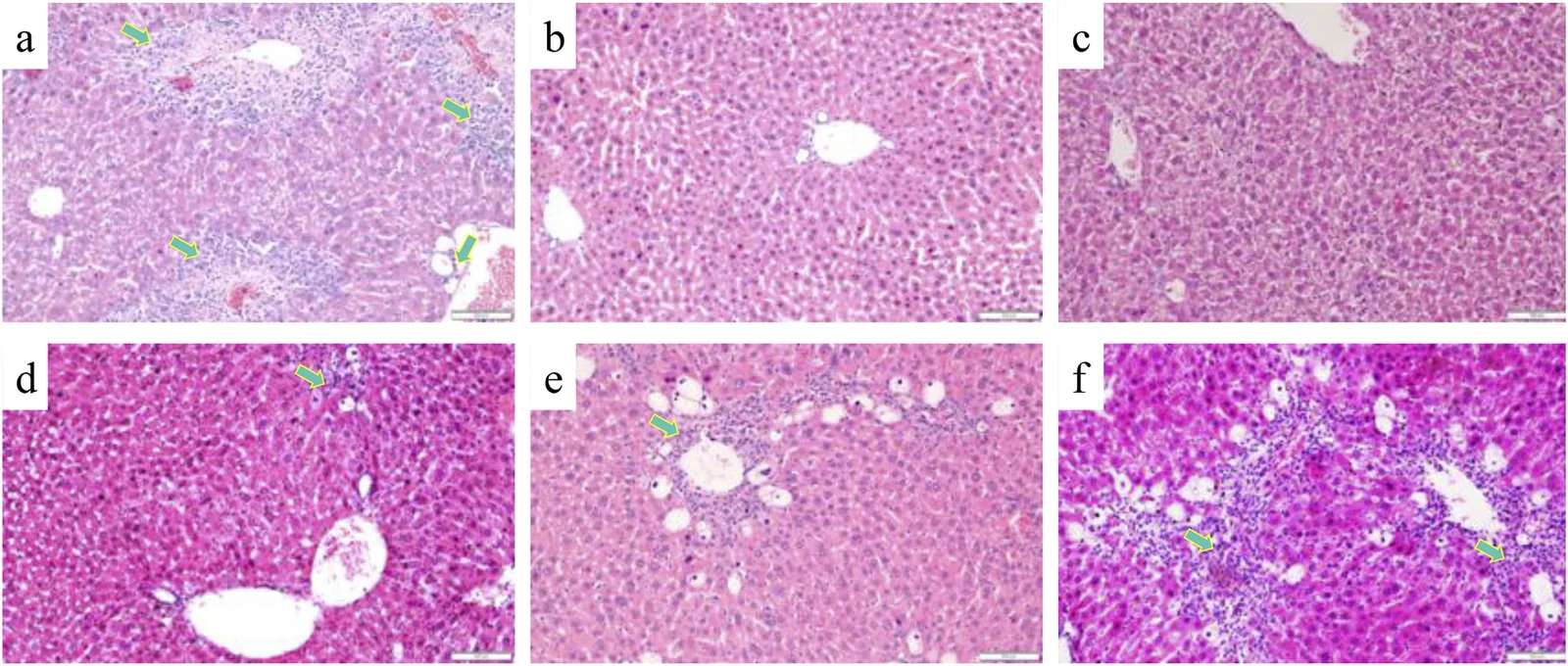
Effect of SP on liver tissue morphology in CCl_4_-induced ALI mice. The sections of liver were stained with H&E and examined at ×200 magnification. Green arrows indicate representative areas of inflammatory lesions characterized by hepatocyte injury and necrosis. **(a)** Model group: Characterized by massive centrilobular necrosis (indicated by green arrows), severe hydropic degeneration with pale, loose pink cytoplasm, marked dark blue/purple inflammatory cell infiltration, and bright red congestion. Notably, no obvious vacuolar degeneration (steatosis) is observed in this severe acute phase. **(b)** Normal group: Exhibits intact hepatic lobular architecture with uniformly eosinophilic pink cytoplasm, centrally located round nuclei, and clear hepatic sinusoids. No evidence of necrosis, inflammation, or steatosis is observed. **(c)** Positive control group: Histological features closely resemble those of the normal group, exhibiting dense pink cytoplasm and intact cords, with no necrotic foci or lipid vacuoles present. **(d)** High-dose group: Displays marked recovery, with rare clear round vacuoles and minimal focal necrosis (occasionally indicated by green arrows). **(e)** Medium-dose group: Demonstrates partial amelioration. Prominent clear, unstained round vacuoles (macrovesicular steatosis) are evident, accompanied by necrotic foci (indicated by green arrows) and moderate dark blue/purple inflammatory infiltration. **(f)** Low-dose group: Exhibits incomplete recovery, characterized by extensive clear vacuoles (steatosis), obvious necrotic areas (indicated by green arrows), prominent dark blue/purple inflammatory infiltration, and focal red congestion. The quantitative analysis of necrotic area percentage is presented in Table 1.

Quantitative histopathological analysis of the liver sections (Figure 5) revealed that the necrotic area percentage varied markedly across groups. The model group exhibited the highest necrotic area (23.57 ± 0.82)%, while the normal group showed minimal necrosis (0.01 ± 0.02)%. Treatment with SP reduced the necrotic area in a dose-dependent manner: high-dose SP (0.76 ± 0.08)%, medium-dose SP (6.42 ± 0.26)%, and low-dose SP (17.34 ± 1.09)%. The positive control group showed a necrotic area of 0.19% ± 0.02%, comparable to the high-dose SP group. These data indicate that SP exerts a dose-dependent protective effect against CCl_4_-induced hepatic necrosis (Table 1).

**TABLE 1:** Quantitative analysis of necrotic area percentage in liver tissues from each group.

Groups	Dosage	Necrotic area percentage (%, mean ± SD)
Normal group	0.2 ml	-
Model group	0.2 ml	23.57 ± 0.82
Positive group	150 mg/kg	0.19 ± 0.02
SP high group	2 g/kg	0.76 ± 0.08
SP midium group	1 g/kg	6.42 ± 0.26
SP low group	0.5 g/kg	17.34 ± 1.09

### 3.6 Effects of SP on hepatic oxidative stress in CCL4-induced ALI mice

Compared with the normal group, SOD activity (Figure 6a) was significantly decreased, while MDA levels (Figure 6b) were significantly raised in the model group (*p* < 0.01, *p* < 0.05), indicating enhanced oxidative stress. Treatment with SP significantly increased SOD activity (*p* < 0.05) and markedly reduced MDA levels (*p* < 0.01) relative to the model group, demonstrating that SP mitigates CCl_4_-induced oxidative damage in liver tissue.

**FIGURE 6:**
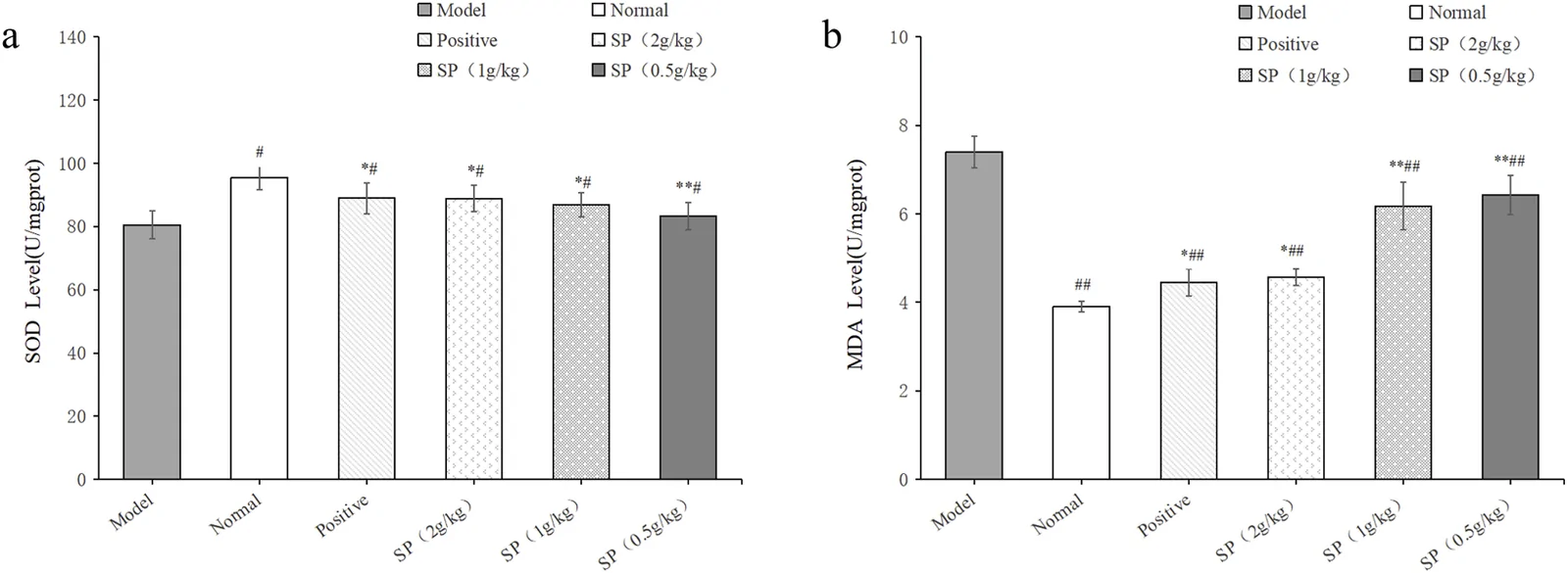
Effects of SP on hepatic oxidative stress in CCl_4_-induced ALI mice. Hepatic levels of **(a)** SOD, and **(b)** MDA. ^##^ *p* < 0.01, ^#^ *p* < 0.05 compared with the model group; **p < 0.01, *p < 0.05 vs. normal group.

### 3.7 Effects of SP on hepatocyte mitochondria–ER ultrastructure in CCl_4_-induced ALI mice

TEM analysis revealed severe ultrastructural abnormalities in hepatocytes of the model group, including shrunken nuclei, swollen mitochondria with deformed or absent cristae, dilated and disorganized ER with vacuolization, and extensive ribosomal detachment (Figure 7a). In comparison, hepatocytes in the normal control group displayed normal morphology, with round and intact nuclei, abundant mitochondria with clear cristae, and well-organized ER with attached ribosomes (Figure 7b). In the positive control group, nuclei remained intact with uniform chromatin distribution; mitochondria showed mild swelling, and the ER was largely intact with minor ribosomal detachment and occasional lipid droplets (Figure 7c).

**FIGURE 7:**
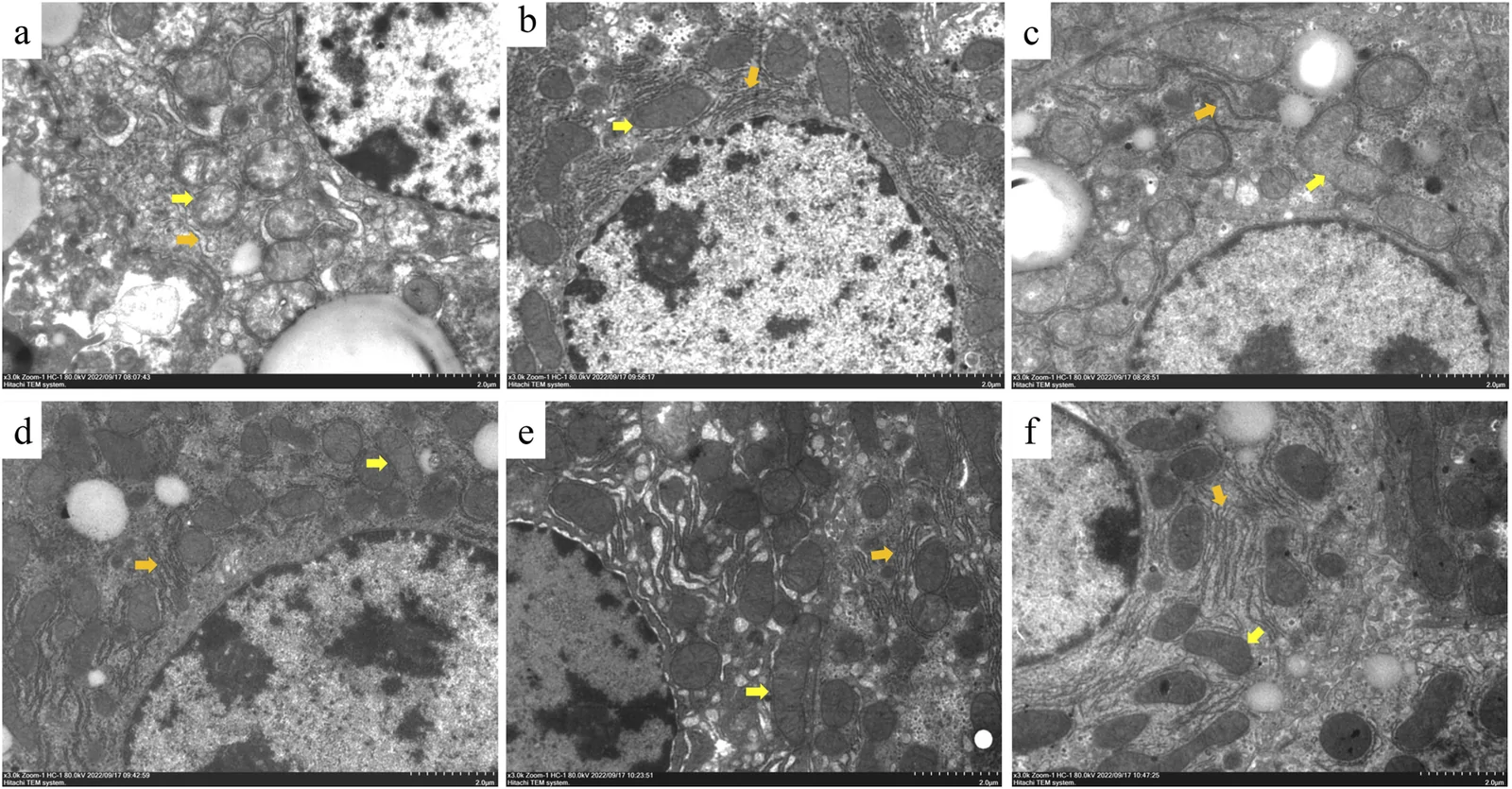
Ultrastructural changes in liver tissues in CCl_4_-induced ALI mice. **(a)** Model group. **(b)** Normal control group. **(c)** Positive control group. **(d)** High-dose SP group. **(e)** Medium-dose SP group. **(f)** Low-dose SP group. Magnification ×3,000. Yellow arrows indicate mitochondria, and orange arrows indicate ER. Following treatment with SP, the ultrastructural integrity of mitochondria and endoplasmic reticulum in hepatic tissues showed marked improvement, indicating attenuation of CCl_4_-induced cellular damage.

In the high-dose SP group, nuclear morphology was slightly altered, while mitochondria and ER structures largely resembled normal hepatocytes, though a few mitochondria and ER remained swollen. The quantity of mitochondria and ER was markedly higher than in the model group (Figure 7d). Medium-dose SP treatment resulted in mild nuclear abnormalities, partially disrupted mitochondrial cristae, and modest expansion of the ER with limited ribosomal detachment (Figure 7e). In the low-dose SP group, nuclear morphology was relatively preserved, but mitochondria were swollen with blurred cristae, ribosomal detachment was observed, and lipid droplets were present (Figure 7f).

### 3.8 Effects of SP on GRP78/IRE1α/JNK CCL_4_-induced ALI mice

Western blot analysis (Figure 8a) demonstrated that SP inhibited activation of the GRP78/IRE1α/JNK signaling pathway in liver tissue. Compared with the model group, treatment with bifendate or SP significantly downregulated GRP78 (*p* < 0.01, *p* < 0.05; Figure 8b), IRE1α (*p* < 0.01, *p* < 0.05; Figure 8c), and JNK (*p* < 0.01, *p* < 0.05; Figure 8d), indicating suppression of ER stress–mediated signaling.

**FIGURE 8:**
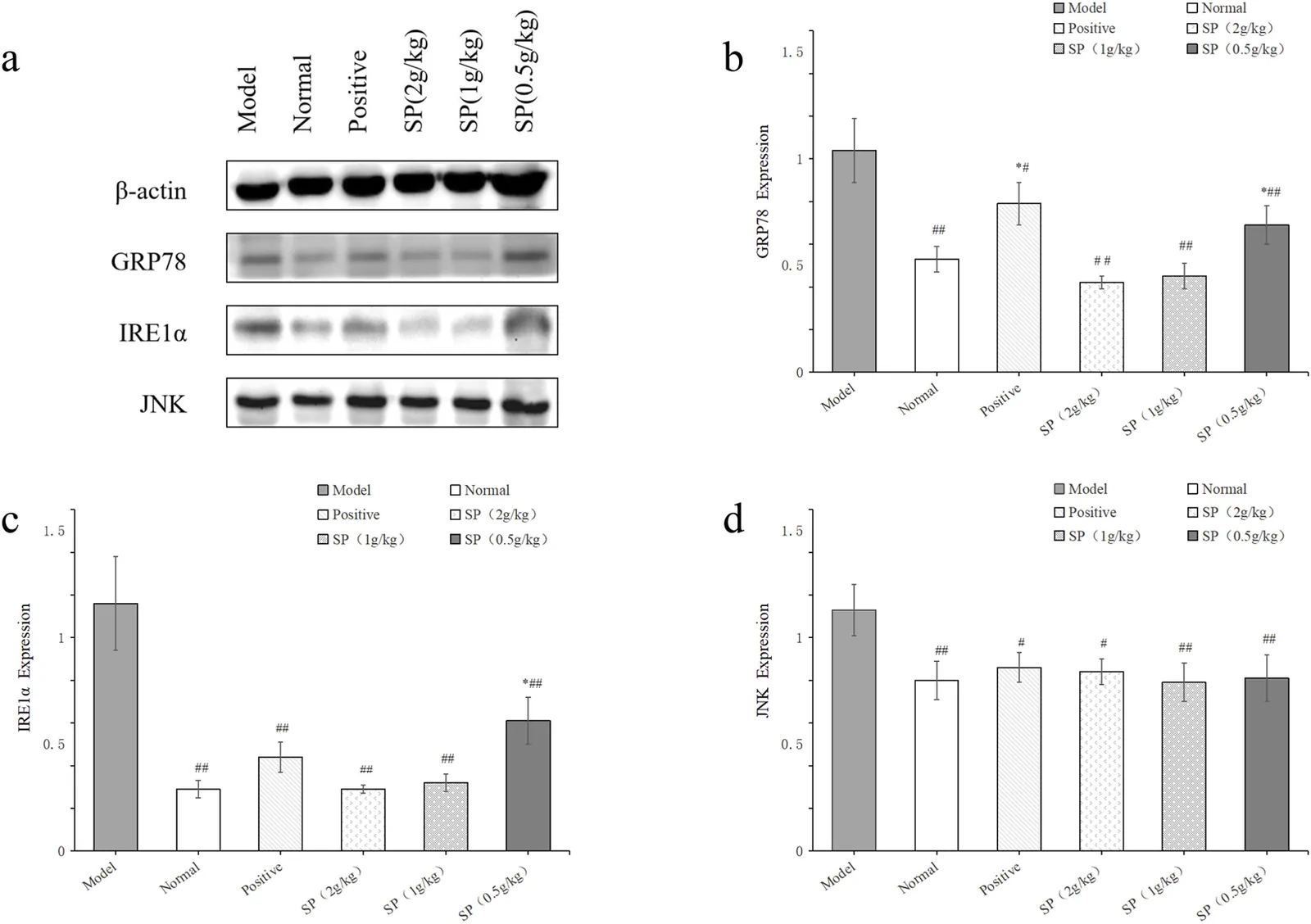
Effects of SP on the GRP78/IRE1α/JNK pathway in liver tissues of CCl_4_-induced ALI mice. **(a)** Representative Western blots showing GRP78, IRE1α, and JNK expression, with β-actin as the loading control. **(b–d)** Densitometric quantification of GRP78 (b), IRE1α **(c)**, and JNK **(d)** expression. SP treatment downregulated the protein expression of GRP78, IRE1α, and JNK compared with the model group. ^##^ *p* < 0.01, ^#^ *p* < 0.05; **p < 0.01, *p < 0.05 vs. normal group.

## 4 Discussion

ALI is a life-threatening disease, often resulting from drug toxicity, viral infection, or acute fatty liver (Yu et al., 2023). Without effective intervention, it may progress to hepatitis or cirrhosis and even result in liver failure (Sun et al., 2022). In TCM, the liver corresponds to the wood element, which thrives on smoothness and is adversely affected by depression. Therapeutic approaches focus on regulating the liver, strengthening the spleen, promoting qi circulation, and resolving blood stasis (Qu et al., 2024). Accumulating evidence indicates that TCM formulations and their bioactive constituents can attenuate liver injury primarily through the suppression of oxidative stress (Hu et al., 2021) and inflammatory responses (Li et al., 2025). Ba Zhen Tang (Eight-Treasure Decoction) has been reported to inhibit the expression of pro-inflammatory mediators, including iNOS, COX-2, TNF-α, NF-κB, IL-1β, and IL-6, while restoring antioxidant enzyme activities such as SOD, CAT, GR, and GPx, enhancing antioxidant and anti-inflammatory capacity and exerting hepatoprotective effects (Song et al., 2014). Xiaoyao San exhibits anti-inflammatory, antioxidant, and anti-apoptotic properties and downregulates p-JAK2 and p-STAT3 protein levels, inhibiting activation of the JAK2–STAT3 signaling pathway in CCl_4_-induced acute liver injury (Zhang et al., 2022). *Lonicera japonica* (honeysuckle) extract ameliorates CCl_4_-induced acute liver injury in rats via activation of the Nrf2 signaling pathway (Lu et al., 2022). All three extracts of *Phragmites communis* (Lu Gen) have been shown to exert protective effects against acute liver injury in rats through antioxidant activity and improvement of lipid metabolism (Rao et al., 2022).

Modern research has demonstrated that SP exerts a hepatoprotective effect by modulating the biosynthetic pathways of tyrosine, phenylalanine, and tryptophan, thereby improving metabolic disorders associated with alcohol-induced liver injury (Deng et al., 2021). SP also shows antioxidant and anti-apoptotic properties, which reduce inflammatory cell infiltration and hepatocyte necrosis, promoting the recovery of liver function (Meng et al., 2022).

Unlike previous studies that primarily investigated the antioxidant effects of SP in CCl_4_-induced liver injury (Zhang et al., 2012), the present study provides mechanistic insights into the ER stress pathway and specifically identifies the GRP78/IRE1α/JNK axis as a key target. Furthermore, Chen et al. (2015) demonstrated that calycosin protects against CCl_4_-induced liver injury via activation of FXR and STAT3; however, whether SP acts through the GRP78/IRE1α/JNK pathway has not been previously reported. The novelty of the present study lies in the integrated investigation of SP’s hepatoprotective effects through this pathway, combining biochemical, histopathological, ultrastructural, and molecular analyses. To our knowledge, this is the first study to demonstrate that SP attenuates CCl_4_-induced ALI via suppression of ER stress-mediated signaling through this specific axis. Our findings thus extend the current understanding of ER stress-related mechanisms in CCl_4_-induced ALI and provide a novel mechanistic basis for the hepatoprotective action of SP.

In ALI treatment, some TCM may indirectly induce renal injury by altering renal metabolism or hemodynamics while providing hepatoprotection (Wang et al., 2023). To evaluate drug safety, we performed dose-dependent toxicology experiments on SP before the main study.

The CCl_4_-induced ALI model produced typical biochemical and histopathological alterations, as reported previously (Hou et al., 2024). Notably, SP treatment significantly attenuated these changes, including reduced serum transaminases, improved organ indices, and decreased inflammatory infiltration. These findings are consistent with the hepatoprotective effects observed in other TCM formulations (Song et al., 2014; Zhang et al., 2022), and further support the therapeutic potential of SP in ALI.

Malondialdehyde (MDA) is a key end product of lipid peroxidation. Under oxidative stress, polyunsaturated fatty acids in cell membranes are attacked by reactive oxygen species, initiating a lipid peroxidation cascade that ultimately generates metabolites such as MDA (Devos et al., 2026). Elevated MDA levels have been closely associated with the onset and progression of liver diseases, and increased concentrations have been reported in multiple hepatic disorders, including alcoholic cirrhosis, alcoholic hepatitis, and non-alcoholic fatty liver disease (Zelber-Sagi et al., 2020).

Superoxide dismutase (SOD) is a critical antioxidant enzyme that catalyzes the dismutation of superoxide anion radicals into hydrogen peroxide and oxygen, interrupting lipid peroxidation chain reactions and protecting hepatocytes from oxidative injury (Zhang et al., 2018; Cai et al., 2023). Under oxidative stress, SOD activity is often reduced due to excessive consumption, leading to impaired free radical scavenging capacity and aggravated cellular damage. A reciprocal relationship is typically observed between SOD activity and MDA levels during oxidative stress, where hepatocellular injury is associated with decreased SOD activity and increased MDA accumulation (Singh et al., 2020). Therefore, serum SOD activity and MDA levels serve as reliable indicators of hepatic oxidative damage, and their combined assessment is widely used to evaluate the extent of liver injury and the efficacy of antioxidant interventions (Cao et al., 2018).

SP treatment effectively attenuated oxidative stress by restoring SOD activity and reducing MDA accumulation, indicating its antioxidant capacity. Most parameters measured in this study, including serum ALT/AST, MDA, SOD, and histopathological scores, exhibited dose-dependent improvements, with the high-dose SP group showing the most pronounced effects.

Notably, the high dose of SP (2 g/kg) exhibited the most pronounced modulatory effect on the GRP78/IRE1α/JNK pathway. This dose-dependent response may be attributed to the higher bioavailability of active compounds at this concentration, which facilitates sufficient target engagement. Compared with bifendate, SP showed a comparable or superior effect on reducing ER stress markers (Figure 8). This difference suggests that SP may offer additional benefits through its multi-component synergy in modulating ER stress.

However, JNK expression did not show a strict linear dose-response relationship, as the medium-dose group displayed a reduction comparable to the high-dose group. This may suggest that the JNK pathway is sensitive to SP even at moderate doses, or that other compensatory mechanisms come into play at higher doses. The dose-dependent protective effects of SP were further reflected in the histological and molecular findings. Histological evaluation revealed severe necrosis and hydropic degeneration in the CCl_4_ model group. The low-dose group showed partial improvement with residual necrosis, inflammation, and steatosis. The medium-dose group effectively alleviated necrosis and inflammation but retained prominent macrovesicular steatosis. The high-dose group restored near normal hepatic morphology, with only minimal residual changes. Molecularly, low-dose treatment failed to significantly downregulate GRP78, IRE1α, and JNK, whereas medium- and high-dose treatments significantly suppressed these markers, with medium-dose levels approaching those of the high-dose group. These data suggest that the medium dose is sufficient to block ER stress-mediated necrosis and inflammation. It is plausible that the residual steatosis in the medium-dose group indicates that while the molecular inhibition of ER stress rapidly blocks cell death, the restoration of ER-mediated lipid secretion requires a higher dose or a longer duration, which warrants further investigation. In contrast, the high dose achieves comprehensive repair of both signaling and metabolic functions. Collectively, the compound exerts dose-dependent protection against CCl_4_-induced liver injury via inhibition of the ER stress-IRE1α-JNK pathway.

ERS is a signaling pathway activated when intracellular homeostasis is disrupted. Conditions such as protein misfolding, accumulation of unfolded proteins, excessive protein synthesis, or calcium ion imbalance trigger the ER to initiate a stress response aimed at restoring cellular homeostasis (Liu et al., 2024). Free radicals generated from CCl_4_ metabolism can covalently bind to phospholipids in the ER membrane, inducing lipid peroxidation. This process damages the membrane structure and further promotes ERS (Fang et al., 2022). In this study, TEM analysis of the model group showed nuclear shrinkage, marked mitochondrial swelling, and deformation or loss of cristae. SP treatment improved organelle morphology in a dose-dependent manner, with the high-dose group showing minimal ER swelling and near-normal structure, indicating that SP protects ER integrity and mitigates CCl_4_-induced liver injury.

GRP78 is a molecular chaperone that maintains intracellular homeostasis by binding unfolded or misfolded proteins (Ismael et al., 2021; Zheng and Fu, 2021; Amaresan and Gopal, 2023). Inositol-requiring enzyme 1 (IRE1α), a key ERS regulator, possesses kinase and endonuclease activities and contributes to cell fate, immune regulation, and metabolism (Gómora-García et al., 2021). Downregulation of GRP78 and IRE1α can suppress CCl_4_-induced ERS and hepatocyte apoptosis (Liao et al., 2018). In this study, SP significantly reduced GRP78 and IRE1α expression, suggesting that its hepatoprotective effects may be mediated at least in part, through inhibition of ERS *via* the GRP78 and IRE1α pathways.

The JNK signaling pathway mediates cell apoptosis through multiple mechanisms (Zhu L et al., 2024). In ALI, excessive JNK activation can trigger hepatocyte death and inflammatory responses, worsening liver injury (Zhang et al., 2023). Studies indicate that JNK contributes to liver damage by phosphorylating the transcription factor c-Jun in response to external stimuli, promoting the expression of apoptosis-related genes and driving cell death (Devaiah et al., 2024). Abnormal JNK activation also induces upregulation of inflammatory factors, intensifying hepatic inflammation (Wu et al., 2022). In the present study, JNK expression in ALI mice was significantly elevated. SP administration markedly reduced JNK levels compared to the model group, suggesting that SP may exert hepatoprotective effects, possibly by modulating the JNK signaling pathway. This reduction correlated with decreased ALT and AST levels, indicating a potential relationship among JNK, ALT, and AST in the mechanism by which SP alleviates ALI. It is therefore proposed that the JNK pathway may influence ALT and AST levels, contributing to SP’s hepatoprotective effect.

Several limitations of the present study should be acknowledged. First, although the CCl_4_-induced acute liver injury model is widely used, a single intraperitoneal injection does not fully recapitulate the chronic and progressive characteristics of human liver diseases, such as hepatitis or cirrhosis. Second, while the present findings suggest that SP modulates the GRP78/IRE1α/JNK signaling pathway, the precise molecular mechanisms remain unclear, including whether SP constituents directly interact with these proteins or exert their effects through upstream regulatory targets. Third, only three dosage levels of SP were evaluated; therefore, the optimal therapeutic range and potential dose-dependent toxicity outside the tested concentrations were not determined. Fourth, we did not assess the PERK/eIF2α/ATF4/ATF6/CHOP cascade, which represents another major branch of the UPR pathway; this should be addressed in future studies. Fifth, we did not perform HPLC analysis to quantify the active compounds in the SP decoction, which limits the reproducibility and comparability of our findings with other studies. Sixth, this study was conducted under controlled experimental conditions in animal models, and further clinical studies are required to assess the efficacy and safety of SP in human acute liver injury. Finally, our sample size (*n* = 8 per group) was relatively small, and generalizability of the results. Future investigations should incorporate chronic liver injury models, include both sexes, and extend observation periods to improve translational relevance.

In conclusion, this study provides novel evidence that SP exerts significant hepatoprotective effects in CCl_4_-induced ALI through the GRP78/IRE1α/JNK pathway. SP improves liver function and histopathology by reducing serum ALT and AST levels, mitigating oxidative stress, and restoring antioxidant capacity. Mechanistically, SP appears to alleviates hepatocyte injury through modulation of ERS and apoptosis, with the GRP78/IRE1α/JNK signaling pathway, likely playing a role. These findings provide a scientific basis for the potential therapeutic application of SP in ALI and highlight the need for further studies to explore additional molecular mechanisms involved in its hepatoprotective action.

## Data Availability

The original contributions presented in the study are included in the article/supplementary material, further inquiries can be directed to the corresponding author.

## References

[B1] Amaresan R. Gopal U. (2023). Cell surface GRP78: a potential mechanism of therapeutic resistant tumors. Cancer Cell Int. 23 (1), 100. 10.1186/s12935-023-02931-9 37221596 PMC10204160

[B2] Billerbeck E. Wolfisberg R. Fahnøe U. Xiao J. W. Quirk C. Luna J. M. (2017). Mouse models of acute and chronic hepacivirus infection. Science 357 (6347), 204–208. 10.1126/science.aal1962 28706073 PMC5654634

[B3] Breu A. C. Patwardhan V. R. Nayor J. Ringwala J. N. Devore Z. G. Ganatra R. B. (2019). A multicenter study into causes of severe acute liver injury. Clin. Gastroenterol. Hepatol. 17 (6), 1201–1203. 10.1016/j.cgh.2018.08.016 30103039

[B4] Cai L. Zhang S. Huang W. Zhang L. Cai Y. Zhou L. (2023). Oxidative stress and its correlation with nuclear factor-κB and transforming growth factor-β1 in liver injury induced by different doses of X-rays in mice. J. Environ. Occup. Med. 40 (2), 202–208. 10.11836/JEOM22319

[B5] Cao L. Du J. Jia R. Yin G. Jeney G. Ding W. (2018). The liver protective effect of curcumin combined with glycyrrhizic acid and lentinan on CCl_4_-induced hepatic damage in common carp (*Cyprinus carpio*). Jiangsu J. Agric. Sci. 34 (5), 1100–1106. 10.3969/j.issn.1000-4440.2018.05.019

[B6] Chen X. Meng Q. Wang C. Liu Q. Sun H. Huo X. (2015). Protective effects of calycosin against CCl_4_-induced liver injury with activation of FXR and STAT3 in mice. Pharm. Res. 32 (2), 538–548. 10.1007/s11095-014-1483-3 25143196

[B7] Chinese Pharmacopoeia Commission (2020). Pharmacopoeia of the People's Republic of China. Beijing: China Medical Science Press.

[B8] Deng J. Li L. Lin L. Li Y. Xia B. Liao D. (2021). Metabolic mechanism of *Prunella vulgaris* in treatment of ethanol-induced oxidative stress in rats based on metabonomics. China J. Chin. Mat. Med. 46 (7), 1813–1821. 10.19540/j.cnki.cjcmm.20210122.503 33982486

[B9] Devaiah B. N. Singh A. K. Mu J. Chen Q. Meerzaman D. Singer D. S. (2024). Phosphorylation by JNK switches BRD4 functions. Mol. Cell 84 (22), 4282–4296.e4287. 10.1016/j.molcel.2024.09.030 39454579 PMC11585421

[B10] Devos L. Berghe T. V. Monbaliu D. Jochmans I. (2026). Dynamic profile of malondialdehyde in renal and hepatic ischemia reperfusion injury: an explorative study of internal historical samples. bioRxiv.

[B11] Fang C. Zhang W. Zhang J. Qu N. Cao Z. Pan J. (2022). Research status of the construction and application of common liver injury animal models. Chin. J. Clin. Pharmacol. 38 (3), 276–280. 10.13699/j.cnki.1001-6821.2022.03.020

[B12] Gao X. Li S. Liu X. Cong C. Zhao L. Liu H. (2021). Neuroprotective effects of tiaogeng decoction against H_2_O_2_-induced oxidative injury and apoptosis in PC12 cells via Nrf2 and JNK signaling pathways. J. Ethnopharmacol. 279, 114379. 10.1016/j.jep.2021.114379 34216727

[B13] Gómora-García J. C. Gerónimo-Olvera C. Pérez-Martínez X. Massieu L. (2021). IRE1α RIDD activity induced under ER stress drives neuronal death by the degradation of 14-3-3 θ mRNA in cortical neurons during glucose deprivation. Cell Death Discov. 7 (1), 131. 10.1038/s41420-021-00518-9 34083523 PMC8175356

[B14] Hou Z. Tan Z. Xu J. (2024). Regulation effect of dachaihu decoction on inflammatory response in CCl_4_-induced acute liver injury in rats. J. Emerg. Tradit. Chin. Med. 33 (10), 1718–1722. 10.3969/j.issn.1004-745X.2024.10.006

[B15] Hu Q. Zhang W. Wu Z. Tian X. Xiang J. Li L. (2021). Baicalin and the liver-gut system: pharmacological bases explaining its therapeutic effects. Pharmacol. Res. 165, 105444. 10.1016/j.phrs.2021.105444 33493657

[B16] Huang H. Pang X. Dai W. Huang M. Zeng C. (2024). Chaihu shugan granules improve tetrachloromethane-induced acute liver injury in mice through regulating the Keap1-Nrf2/HO-1 signaling pathway. Chin. Pharm. J. 59 (8), 703–712. 10.11669/cpj.2024.08.006

[B17] Ismael S. Sakata K. McDonald M. P. Liao F. F. Ishrat T. (2021). ER stress associated TXNIP-NLRP3 inflammasome activation in hippocampus of human alzheimer's disease. Neurochem. Int. 148, 105104. 10.1016/j.neuint.2021.105104 PMC9479581 34153352

[B18] Li H. Bai G. Ge Y. Zhang Q. Kong X. Meng W. (2018). Hydrogen-rich saline protects against small-scale liver ischemia-reperfusion injury by inhibiting endoplasmic reticulum stress. Life Sci. 194, 7–14. 10.1016/j.lfs.2017.12.022 29253502

[B19] Li S. Li Z. Li H. Liu Z. Liu Y. (2025). Research progress on protective effects of Chinese herbal extracts, active ingredients, and compounds on alcoholic liver injury. Chin. Tradit. Pat. Med. 47 (1), 139–147.

[B20] Liao X. Wu L. Lin Z. Ye J. (2018). Lycopene protects against CCl_4_-induced acute hepatic injury via inhibiting endoplasmic reticulum stress. Chin. J. Clin. Pharmacol. Ther. 23 (7), 770–775.

[B21] Liu S. Zhang X. Yao X. Wang G. Huang S. Chen P. (2024). Mammalian IRE1α dynamically and functionally coalesces with stress granules. Nat. Cell Biol. 26 (6), 917–931. 10.1038/s41556-024-01418-7 38714852

[B22] Lu R. Yang Q. Zhao L. (2022). Protective effect of *Lonicera japonica* extract on liver function in acute liver injury model rats by activating the Nrf2 pathway. Chin. J. Gerontol. 42 (19), 4811–4815.

[B23] Meng W. Liu H. Zhang W. Ma Z. Deng Z. Zhao D. (2022). Research progress on mechanism of traditional Chinese medicine in preventingand treating alcoholic liver disease. Chin. Tradit. Herb. Drugs 53 (3), 868–881. 10.7501/j.issn.0253-2670.2022.03.028

[B24] Mir R. H. Bhat M. F. Sawhney G. Kumar P. Andrabi N. I. Shaikh M. (2022). *Prunella vulgaris* L: critical pharmacological, expository traditional uses and extensive phytochemistry: a review. Curr. Drug Discov. Technol. 19 (1), e140122191102. 10.2174/1570163818666210203181542 33538676

[B25] Qu Q. Li Y. Li Y. (2024). Translation and Introduction of Emergency Prescriptions Worth a Thousand Gold for Ready Reference. Beijing: People's Medical Publishing House.

[B26] Rao Z. Chen G. Xie M. Chen F. Chen Y. Jiang X. (2022). Study on the protective effect of rhizoma phragmitis extracts on acute alcoholic liver injury in rats. Lishizhen Med. Mat. Med. Res. 33 (1), 95–98.

[B27] Shi X. Yang W. Wang N. Zhu J. (2019). Circulating JNK pathway-associated phosphatase level correlates with decreased risk, activity, inflammation level and reduced clinical response to tumor necrosis factor-α inhibitor in crohn disease patients. Med. Baltim. 98 (33), e16622. 10.1097/md.0000000000016622 31415355 PMC6831387

[B28] Singh L. Majhi S. Pabreja K. Negi P. Goyal R. Gupta G. (2020). “Oxidative stress in liver diseases,” in Role of Oxidative Stress in Pathophysiology of Diseases. Editors Maurya P. Dua K. (Singapore: Springer), 209–228.

[B29] Song E. Fu J. Xia X. Su C. Song Y. (2014). Bazhen decoction protects against acetaminophen induced acute liver injury by inhibiting oxidative stress, inflammation and apoptosis in mice. PLoS One 9 (9), e107405. 10.1371/journal.pone.0107405 25222049 PMC4164650

[B30] Song J. Hao M. Zhai X. Chen J. Gui S. (2023). Protective effect of polysaccharides from Platycodon grandiflorum on CCl_4_-induced acute liver injury in mice and its regulatory mechanism. Cent. South Pharm. 21 (1), 110–115.

[B31] Stravitz R. T. Lee W. M. (2019). Acute liver failure. Lancet 394 (10201), 869–881. 10.1016/s0140-6736(19)31894-x 31498101 PMC10836844

[B32] Sun Y. K. Zhang Y. F. Xie L. Rong F. Zhu X. Y. Xie J. (2022). Progress in the treatment of drug-induced liver injury with natural products. Pharmacol. Res. 183, 106361. 10.1016/j.phrs.2022.106361 35882295

[B33] Taru V. Szabo G. Mehal W. Reiberger T. (2024). Inflammasomes in chronic liver disease: hepatic injury, fibrosis progression and systemic inflammation. J. Hepatol. 81 (5), 895–910. 10.1016/j.jhep.2024.06.016 38908436 PMC11881887

[B34] Wan X. Xie X. Liu J. Lu Y. (2016). Hepatoprotective effect of oleanolic acid on D-galactosamine-induced acute liver injury in the mice. West China J. Pharm. Sci. 31 (1), 26–29. 10.13375/j.cnki.wcjps.2016.01.008

[B35] Wang G. Liu X. Deng S. Liao W. Tang L. Jiang W. (2023). Research progress on the mechanism of traditional Chinese medicine against acute liver injury. J. Guangxi Med. Univ. 40 (11), 1921–1927. 10.16190/j.cnki.45-1211/r.2023.11.023

[B36] Wang J. Jiang B. Yang Y. Sun J. Sun Z. Wang P. (2025). Protective effects of extract of *Atractylodes japonica* koidz. ex kitam on carbon tetrachloride-induced acute liver injury in mice. Acta Chin. Med. Pharm. 53 (2), 25–29. 10.19664/j.cnki.1002-2392.250026

[B37] Wu J. Luo F. L. Xie Y. Xiong H. Gao Y. Liu G. (2022). EST12 regulates myc expression and enhances anti-mycobacterial inflammatory response via RACK1-JNK-AP1-Myc immune pathway. Front. Immunol. 13, 943174. 10.3389/fimmu.2022.943174 36003390 PMC9393728

[B38] Yu C. Chen P. Miao L. Di G. (2023). The role of the NLRP3 inflammasome and programmed cell death in acute liver injury. Int. J. Mol. Sci. 24 (4), 3067. 10.3390/ijms24043067 36834481 PMC9959699

[B39] Yuan R. Tao X. Liang S. Pan Y. He L. Sun J. (2018). Protective effect of acidic polysaccharide from Schisandra chinensis on acute ethanol-induced liver injury through reducing CYP2E1-dependent oxidative stress. Biomed. Pharmacother. 99, 537–542. 10.1016/j.biopha.2018.01.079 29902864

[B40] Zelber-Sagi S. Ivancovsky-Wajcman D. Fliss-Isakov N. Hahn M. Webb M. Shibolet O. (2020). Serum malondialdehyde is associated with non-alcoholic fatty liver and related liver damage differentially in men and women. Antioxidants (Basel) 9 (7), 578. 10.3390/antiox9070578 32630732 PMC7401879

[B41] Zhang S. Deng Z. Huang C. Xie J. He Y. Li J. (2012). Protective effects of total triterpenoids of *Prunella vulgaris* L. against carbon tetrachloride-induced acute liver injury in rats. J. Anhui Med. Univ. 47 (9), 1054–1058. 10.3969/j.issn.1000-1492.2012.09.011

[B42] Zhang C. Wang N. Xu Y. Tan H. Y. Li S. Feng Y. (2018). Molecular mechanisms involved in oxidative stress-associated liver injury induced by Chinese herbal medicine: an experimental evidence-based literature review and network pharmacology study. Int. J. Mol. Sci. 19 (9), 2745. 10.3390/ijms19092745 30217028 PMC6165031

[B43] Zhang W. Ge B. Zhou Y. Que R. (2022). Protective effect and mechanism of xiaoyao powder on CCl4-induced acute liver injury. Mod. J. Integr. Tradit. Chin. West. Med. 31 (7), 899–904. 10.3969/j.issn.1008-8849.2022.07.005

[B44] Zhang K. Zhang M. X. Meng X. X. Zhu J. Wang J. J. He Y. F. (2023). Targeting GPR65 alleviates hepatic inflammation and fibrosis by suppressing the JNK and NF-κB pathways. Mil. Med. Res. 10 (1), 56. 10.1186/s40779-023-00494-4 38001521 PMC10675918

[B45] Zheng Z. Fu Q. (2021). Expression and clinical significance of glucose regulatory protein 78 and miR-181-5p in peripheral blood of patients with acute pancreatitis. J. Clin. Med. Pract. 25 (23), 77–81. 10.7619/jcmp.20212089

[B46] Zhu L L. Xie Z. Yang G. Zhou G. Li L. Zhang S. (2024). Stanniocalcin-1 promotes PARP1-dependent cell death via JNK activation in colitis. Adv. Sci. (Weinh.) 11 (5), e2304123. 10.1002/advs.202304123 38088577 PMC10837357

[B47] Zhu S S. Chen S. T. Jin Y. Y. Lu S. W. Zou F. J. Ma W. J. (2024). Analysis and prediction of disease burden of cirrhosis and other chronic liver diseases due to alcohol use in China from 1990 to 2030. Chin. J. Epidemiol. 45 (2), 185–191. 10.3760/cma.j.cn112338-20230920-00173 38413055

